# P-448. Contextual assessment and potential barriers and facilitators of a mobile health program to support treatment of tuberculosis and HIV in Kilimanjaro, Tanzania: opportunities for care integration

**DOI:** 10.1093/ofid/ofae631.648

**Published:** 2025-01-29

**Authors:** Liza Khutsishvili, Carolin Fabian, Kennedy Ngowi, Margaretha Sariko, Krisanta Wilhelm, Stellah Mpagama, Scott Heysell, Marion Sumari-De Boer, Jacqueline Hodges

**Affiliations:** University of Virginia, Charlottesville, Virginia; University of Virginia, Charlottesville, Virginia; Kilimanjaro Clinical Research Institute, Moshi, Kilimanjaro, Tanzania; Kilimanjaro Clinical Research Institute, Moshi, Kilimanjaro, Tanzania; Kilimanjaro Clinical Research Institute, Moshi, Kilimanjaro, Tanzania; Kibong’oto Infectious Diseases Hospital, Moshi, Kilimanjaro, Tanzania; University of Virginia, Charlottesville, Virginia; Kilimanjaro Clinical Research Institute, Moshi, Kilimanjaro, Tanzania; Duke University School of Medicine, Cary, North Carolina

## Abstract

**Background:**

Despite increasing smartphone penetration globally, personalized mobile health (mHealth) care interventions remain largely untapped for people with tuberculosis (TB) in many settings. A smartphone platform adopted to support treatment of human immunodeficiency virus (HIV) in the US was adapted and piloted for HIV/TB care in a distinct urban population, with high usage and improved clinical outcomes. Systematic evaluation of potential contextual influences on mHealth implementation is needed to ensure appropriateness, feasibility, and acceptability of the intervention for HIV/TB care within Kilimanjaro.

**Table 1:**
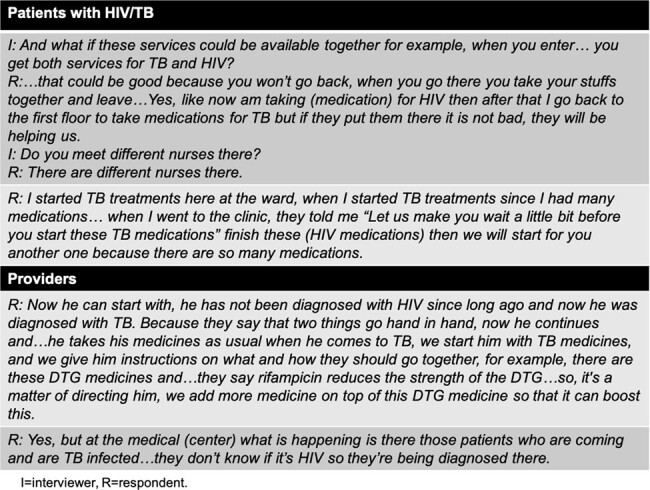
Perspectives on existing HIV and TB care delivery experiences and integration of services

**Methods:**

We conducted semi-structured in-depth interviews at Kilimanjaro Christian Medical Centre and Kibong’oto Hospital with patients (18 y/o+ with drug-susceptible/drug-resistant-TB, with/without HIV, > 1 month on treatment) and providers (e.g. physicians, nurses). Guides were designed using Bury’s Framework for Chronic Illness and the Consolidated Framework for Implementation Research (CFIR). Analysis was performed using *a priori* open coding/constant comparisons methodology. A preliminary codebook built deductively from frameworks was iterated with inductive codes based on emergent themes.

**Results:**

To date, 12 patient and 12 provider interviews have been conducted; analysis is underway. Specific perspectives demonstrated opportunities for enhancement of HIV/TB care integration (**Table 1**). Preliminary analysis suggests feature phone access is more common among patients. Additional themes relate to perceptions of an external intervention source (CFIR domain: Intervention Characteristics), compatibility with current provider smartphone access/workload, existing directive nature of patient-provider relationships, and current level of system digitization at sites (workplace internet stability/data access) (Inner Setting).

**Conclusion:**

Analysis to date reveals several prominent themes to consider for adaptation of the intervention: 1) daily life/experiences with TB/HIV 2) determinants of and 3) experiences with TB/HIV care 4) mobile phone ownership, usage 5) perceptions of the intervention within the context and to support 6) home urine testing for personalized dosing of TB medications.

**Disclosures:**

**Jacqueline Hodges, MD MPH**, Gilead Sciences: Grant/Research Support

